# Combined Anterior Opening-Wedge High Tibial Osteotomy and Tibial Tubercle Osteotomy with Posterior Cruciate Ligament Reconstruction

**DOI:** 10.1016/j.eats.2021.12.014

**Published:** 2022-03-19

**Authors:** Ajay C. Kanakamedala, Aaron Gipsman, Dylan T. Lowe, Eric J. Strauss, Michael J. Alaia

**Affiliations:** New York University Langone Orthopedic Hospital, New York, New York, U.S.A.

## Abstract

Despite multiple advances in techniques for posterior cruciate ligament reconstruction (PCL-R), residual posterior laxity continues to be a commonly reported complication. Multiple studies demonstrated a decreased or flat posterior tibial slope, increases posterior laxity, and forces placed across the native and reconstructed PCL. Anterior opening wedge high tibial osteotomies (aOW-HTO) can be used to increase posterior tibial slope, thereby reducing tibial sag and posterior laxity. Depending on the technique used, anterior opening wedge osteotomies can lead to changes in patellar height, affecting patient pain and satisfaction. The purpose of this article is to describe a technique for an aOW-HTO with a tibial tubercle osteotomy and concomitant PCL-R to increase the posterior tibial slope while minimizing changes to patellar height.

## Introduction

Residual posterior laxity after posterior cruciate ligament reconstruction (PCL-R) remains a clinical challenge, with reported rates ranging from 33 to 56% of patients.^1^^,^^2^ There is growing awareness of the effect of reduced posterior tibial slope (PTS) on posterior knee laxity.^1^^,^^3^, ^4^, ^5^ Reduced PTS leads to increased posterior tibial translation in both extension and flexion, leading to increased forces placed across the native and reconstructed PCL, increased posterior tibial sag, and increased risk of PCL injury and PCL-R failure.^3^^,^^6^^,^^7^ Some authors have suggested using anterior opening wedge high tibial osteotomies (aOW-HTO) to increase PTS and decrease posterior knee laxity in the setting of PCL deficiency.^1^^,^^5^ In this article and associated video (Video 1), we present a technique for a combined aOW-HTO and tibial tubercle osteotomy (TTO) with concomitant PCL-R to increase PTS in the setting of PCL deficiency.

## Surgical Technique

### Indications

**Fig 1:**
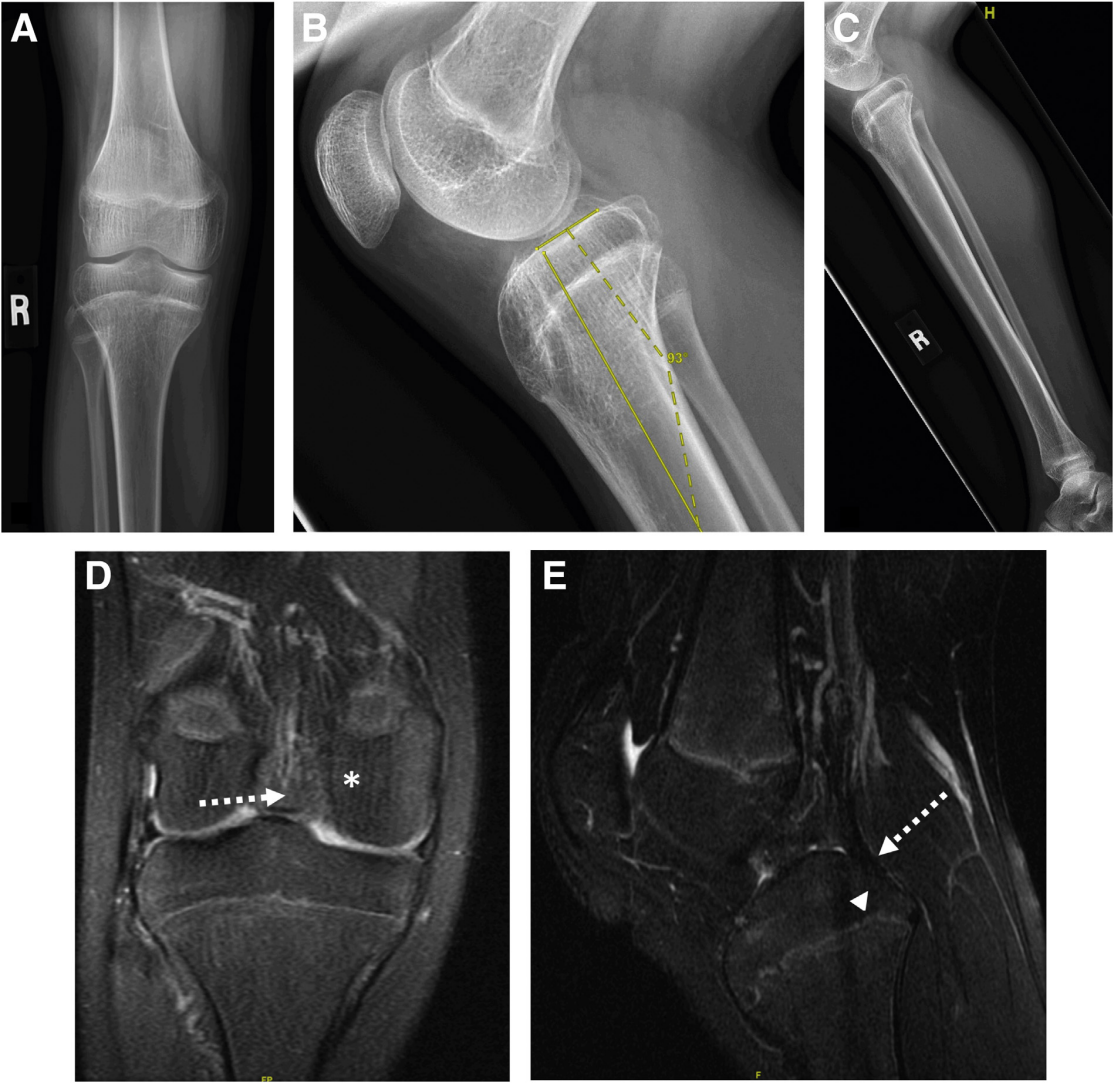
Example of reduced tibial slope. Anteroposterior (A) and lateral (B) radiographs of a right knee demonstrate neutral coronal alignment and tibial slope of −3°. A lateral radiograph of the tibia (C) shows no other deformity in the tibia. Corresponding coronal (D) and sagittal (E) magnetic resonance imaging in this patient shows absent posterior cruciate ligament fibers (dotted white arrows) at their origin on the lateral aspect of the medial femoral condyle (asterisk) and posterior tibial insertion (white arrowhead).

A combined aOW-HTO and TTO is indicated in the setting of symptomatic PCL deficiency with concomitant neutral or negative tibial slope (Fig 1). Posterior laxity should be reassessed intraoperatively after osteotomy fixation with a posterior drawer and posterior stress radiographs, and the decision to perform a PCL-R should be made at that time. A grade II or III posterior drawer or asymmetric posterior tibial translation on posterior stress radiographs after osteotomy fixation suggests significant posterior laxity requiring PCL-R. Advantages and disadvantages of this technique are shown in Table 1.

**Table 1:** Advantages and Disadvantages of the Proposed Technique

Advantages	Disadvantages
Provides significant increase in posterior tibial slope while minimizing change in patellar height	Risk of arthrofibrosis with combined osteotomy and PCL-R
Osteotomy is performed through cancellous bone, which has greater healing potential than an infratubercle osteotomy though cortical bone.	Risk of delayed healing or nonunion
Opening wedge osteotomy alone can potentially obviate the need for PCL-R.	

PCL-R, posterior cruciate ligament reconstruction.

### Patient Setup

Patients are positioned supine on a radiolucent table. A combination of general and regional anesthesia is used. Examination under anesthesia is performed, including examination for posterior laxity with posterior drawer and sag testing, and for concomitant posterolateral corner injuries with dial and external rotation recurvatum testing. A perfect lateral radiograph of the knee at 30° of flexion is obtained for patellar height relative to Blumensaat’s line. A tourniquet is secured around the thigh, and the image intensifier is positioned on the contralateral side. A large sterile bump or radiolucent triangle is used to position the knee at 45-90° of flexion.

### Diagnostic Arthroscopy

Standard diagnostic arthroscopy of the knee is performed to evaluate the status of the cruciate ligaments, articular cartilage, and menisci. Intra-articular chondral and meniscal pathology can be addressed at this time.

### Approach

**Fig 2:**
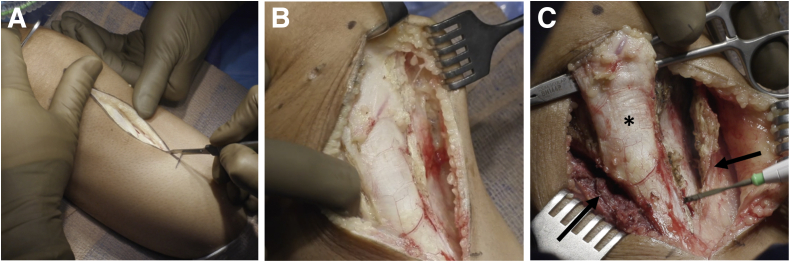
Surgical approach. In a right knee, a 10-cm incision starting just proximal to the tibial tubercle (A) is used. (B) Medial and lateral skin flaps are raised. The patellar tendon (asterisk) is freed from the medial and lateral capsule, a straight clamp is used to define the proximal aspect of the tibial tubercle (black arrowhead), and subperiosteal flaps (black arrows) are raised to expose the proximal tibia (C).

A 10-cm anterior incision is made centered over the tibial tubercle starting just proximal to it, and the lateral and medial full-thickness flaps are raised (Fig 2). Subperiosteal dissection is performed to expose the proximal tibia medially and laterally.

### Tibial Tubercle Osteotomy

**Fig 3:**
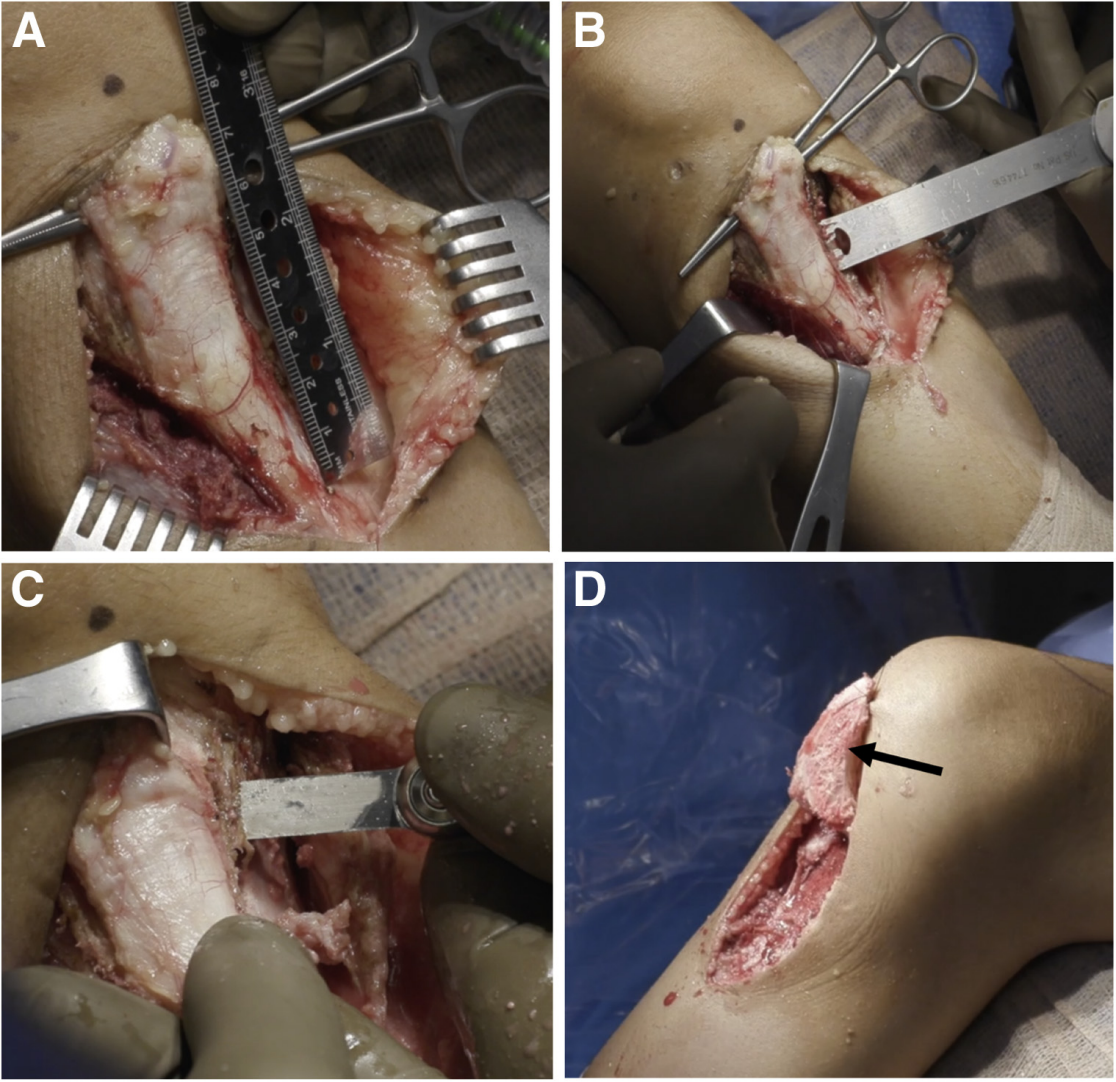
Tibial tubercle osteotomy. After the proximal tibia in a right knee has been exposed medially and laterally, a flat tibial tubercle osteotomy is performed to yield a tibial tubercle fragment approximately 5 cm in length (A and B). (C) A microsagittal saw is used to complete the osteotomy proximally. The osteotomized tibial tubercle (black arrow) is then mobilized proximally, held with a stay suture (D), and covered with a moist towel.

The patellar tendon is released from the medial and lateral capsule (Fig 3). A flat tibial tubercle osteotomy is performed with an oscillating saw to yield an osteotomized tibial tubercle fragment around 5 cm in length. This fragment is mobilized proximally and held with a stay suture proximally.

### High Tibial Osteotomy

**Fig 4:**
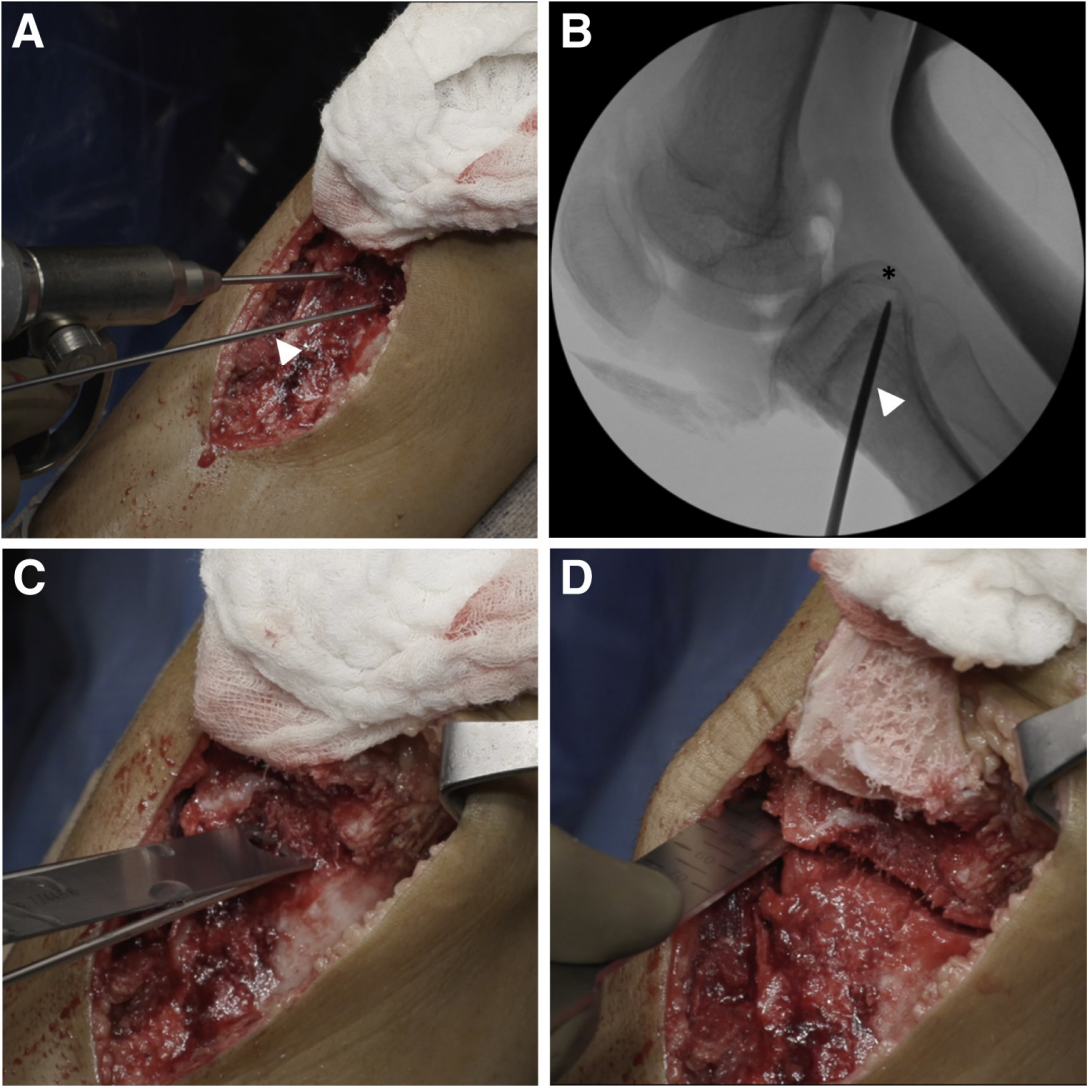
High tibial osteotomy. In a right knee, two guidewires (white arrowheads) are placed (A) to template the high tibial osteotomy under fluoroscopy (B). The guidewires are placed from anterior to posterior and angled distal to proximal. The osteotomy should be distal enough to allow for adequate bone stock proximally for stable fixation of the osteotomy. The posterior hinge of the osteotomy should be distal to the insertion of the PCL on the tibia (asterisk), and proximal enough to be in cancellous bone to maximize bone healing potential. The osteotomy is started with an oscillating saw (C) and finished with an osteotome (D) under fluoroscopic visualization.

Under fluoroscopic visualization, two guidewires are placed from anterior to posterior, angled distal to proximal aiming toward the PCL facet (Fig 4). The osteotomy should be distal enough allowing for adequate bone stock proximally for stable fixation of the osteotomy. The posterior hinge of the osteotomy should be distal to the insertion of the PCL on the tibia, and proximal enough to be in cancellous bone to maximize bony healing potential.

**Fig 5:**
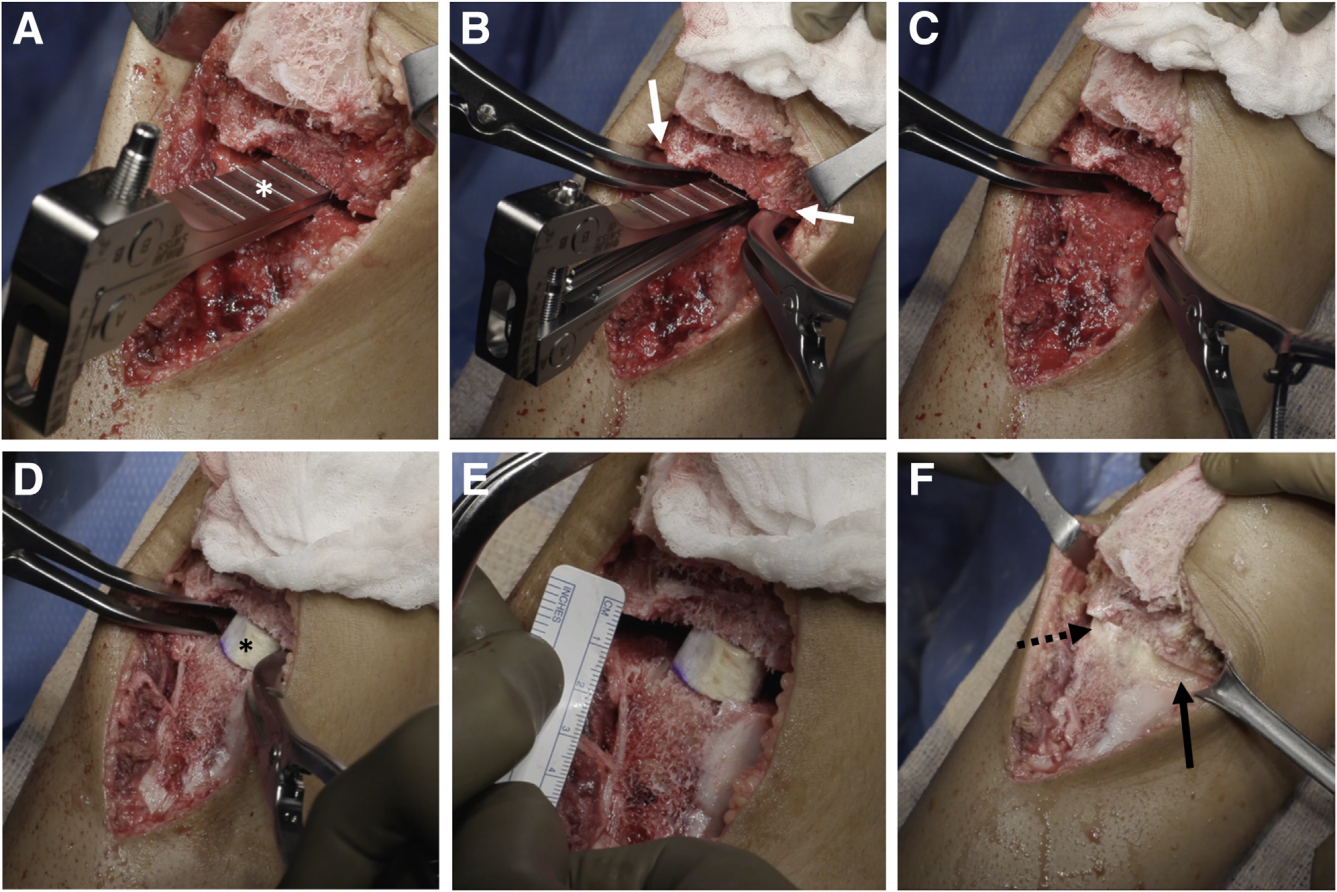
Opening the high tibial osteotomy site. In a right knee, a laminar spreader (white asterisk) is first placed centrally to open the osteotomy site (A). Two laminar spreaders are then placed under the stronger medial and lateral cortices (white arrows, B) and the central laminar spreader is removed (c). A wedge of iliac crest bone allograft (asterisk) is measured, shaped, and inserted into the osteotomy site (D), and the amount of correction is measured (E). Approximately 1 mm of osteotomy site opening corresponds to 1° of correction. Additional bone graft substitutes are packed into the osteotomy site, including demineralized bone matrix (solid black arrow) and a preshaped block of calcium phosphate cement (dotted black arrow, F).

Once the guidewires are in position, an oscillating saw is used to begin the osteotomy, and an osteotome to finish under fluoroscopic visualization. The osteotomy site is slowly opened using a centrally placed laminar spreader (Fig 5). To minimize disruption of the weaker central cancellous bone, two laminar spreaders are then placed on both the medial and lateral cortices to complete the correction. Approximately 1 mm of opening at the osteotomy site corresponds to 1° of correction. Up to 10° of correction can be obtained before the risk of cortical hinge fracture becomes significant. Structural bone graft is measured, trimmed, and placed into the osteotomy site. The authors prefer iliac crest wedge allograft; however, other options include iliac crest autograft or preshaped wedges of calcium phosphate cement.

### Fixation

Fixation can be obtained using specialized precontoured plates for high tibial osteotomies or low profile plates with proximal locking screw capability. If performing the latter, we recommend plating on the medial and lateral sides of the osteotomy. Once fixation is complete, demineralized bone matrix is used to fill the osteotomy site.

Attention is turned to the tibial tubercle. The tibial tubercle fragment is provisionally fixed with Kirschner wires. To avoid any change in patellar height, the tibial tubercle fragment should be proximal to its original location. If the osteotomy site is opened by 1 cm, the tibial tubercle should move approximately 1 cm proximally. A perfect lateral radiograph at 30° is obtained after provisional fixation to assess patellar height and ensure no significant change from preoperative radiographs (Fig 1). Fixation is obtained with two lag screws. The authors’ preference is to use 4.5-mm cannulated fully threaded headless compression screws (Arthrex, Naples, FL).

### Posterior Cruciate Ligament Reconstruction

**Fig 6:**
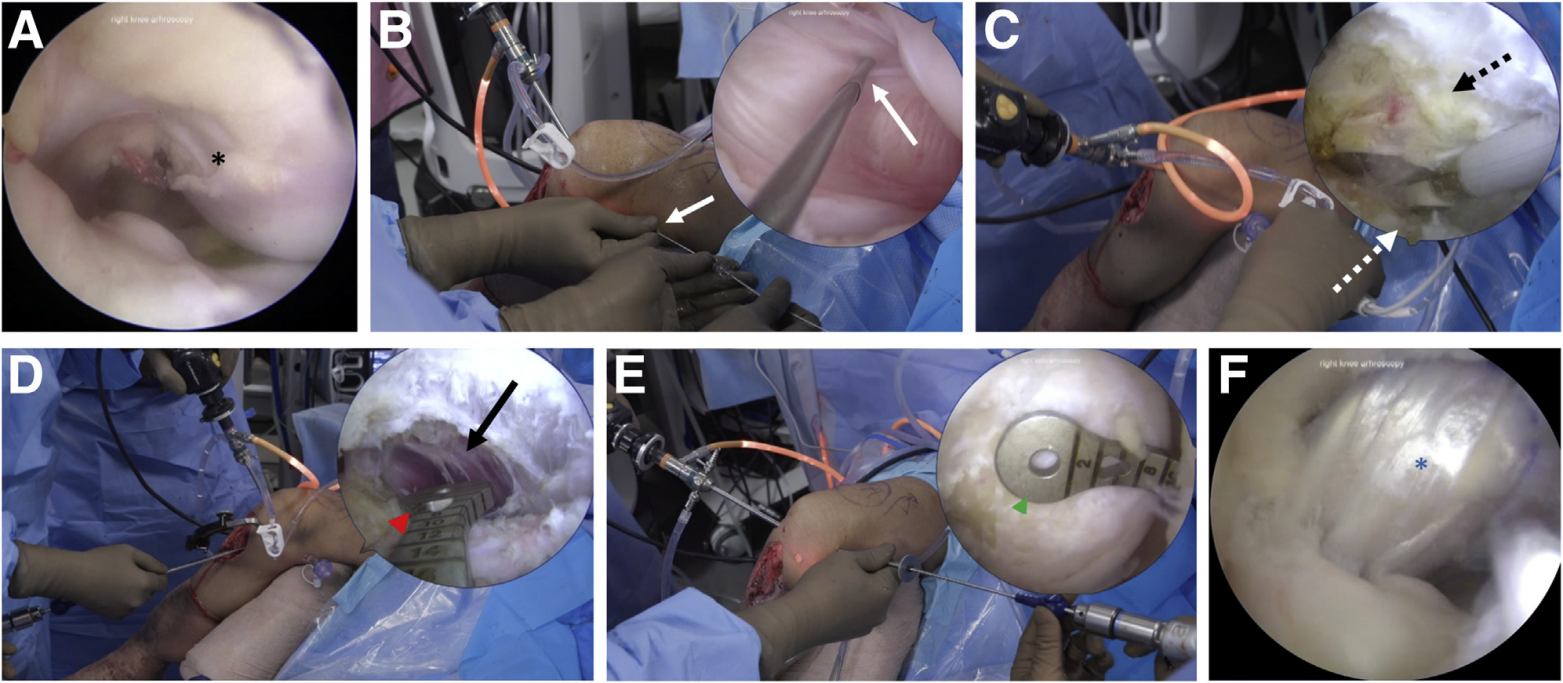
Posterior cruciate ligament (PCL) reconstruction. Diagnostic arthroscopy is performed in a right knee using a 30° arthroscope, unless otherwise noted. (A) Arthroscopy through the anterolateral portal reveals that the PCL is absent from its normal origin (black asterisk) on the medial femoral condyle. (B) Under direct visualization through the anterolateral portal, the posteromedial portal is established (white solid arrows) using a modified Seldinger technique. (C) Using a 70° arthroscope in the anteromedial portal, the surgeon debrides the PCL remnant on the tibia (dotted white arrow), and the posterior capsule (dotted black arrow) is elevated. (D) Once the fibers of the popliteus (black arrow) are visualized with the 70° arthroscope in the anteromedial portal, the tibial tunnel (red arrowhead) is drilled approximately 10-15 mm distal to the articular surface. (E) Under arthroscopic visualization through the anterolateral portal, the femoral tunnel (green arrowhead) is drilled to the anterolateral aspect of the medial femoral condyle. (F) The final PCL allograft (blue asterisk) as seen through the anterolateral portal is shown.

The PCL remnant is debrided through the anteromedial and anterolateral portal. The posteromedial portal is made using a modified Seldinger technique, and a 7-mm cannula is placed (Fig 6). The shaver is reintroduced through the posteromedial portal complete the PCL debridement off of the tibial insertion. The posterior capsule is elevated off of the posterior tibia until the popliteus muscle fibers are visualized. A 70° arthroscope is then used to view the posterior tibial facet through the posteromedial portal. A specialized PCL tibial drilling guide is used (Arthrex, Naples, FL) for the anteromedial portal, and the tibial tunnel is drilled and then back-reamed. A looped shuttling suture is passed through the PCL tibial tunnel for later graft passage. The 30° arthroscope is then used via the anterolateral portal to view the femoral PCL insertion on the lateral aspect of the medial femoral condyle. A femoral guide (Arthrex, Naples, FL) is used to create the femoral tunnel, which is subsequently drilled and back-reamed. The femoral end of the graft is pulled into the femoral tunnel, and the suspensory button is flipped. The tibial end of the graft is then pulled into the tibial tunnel, and the second suspensory button is flipped. The knee is placed at 90° of flexion, an anterior drawer is applied, and the graft is subsequently tensioned on both the femoral and tibial sides.

After the incisions are copiously irrigated, additional bone graft substitute, such as calcium phosphate cement can be placed in the osteotomy site, and a layered closure is performed. Pearls and pitfalls of this technique are shown in Table 2.

**Table 2:** Pearls and Pitfalls of the Proposed Technique

Pearls	Pitfalls
It is important to raise thick subperiosteal flaps that can later be closed to improve healing.	If unevenly sized grafts are used medially and laterally in the osteotomy site, inadvertent change in coronal alignment can occur.
Perfect 30° lateral radiographs should be obtained prior to incision and after provisional TTO fixation to ensure no change in patellar height.	The osteotomy fixation, specifically the proximal most screws, can potentially interfere with reverse reaming of the tibial tunnel. Screws should not be faced toward the tibial PCL facet.
Knee should be flexed during high tibial osteotomy and fixation to minimize risk to posterior neurovascular structures and to facilitate correction.	Opening osteotomy through centrally placed laminar spreader alone can cause disruption of cancellous bone. Place laminar spreaders medially and laterally under stronger cortical bone to complete correction.
Posterior knee laxity should be reassessed after osteotomy fixation to determine whether PCL reconstruction is indicated.	Failure to move tibial tubercle proximal to original location after anterior opening wedge osteotomy leads to iatrogenic patellar baja.

PCL, posterior cruciate ligament; TTO, tibial tubercle osteotomy.

### Postoperative Rehabilitation

**Fig 7:**
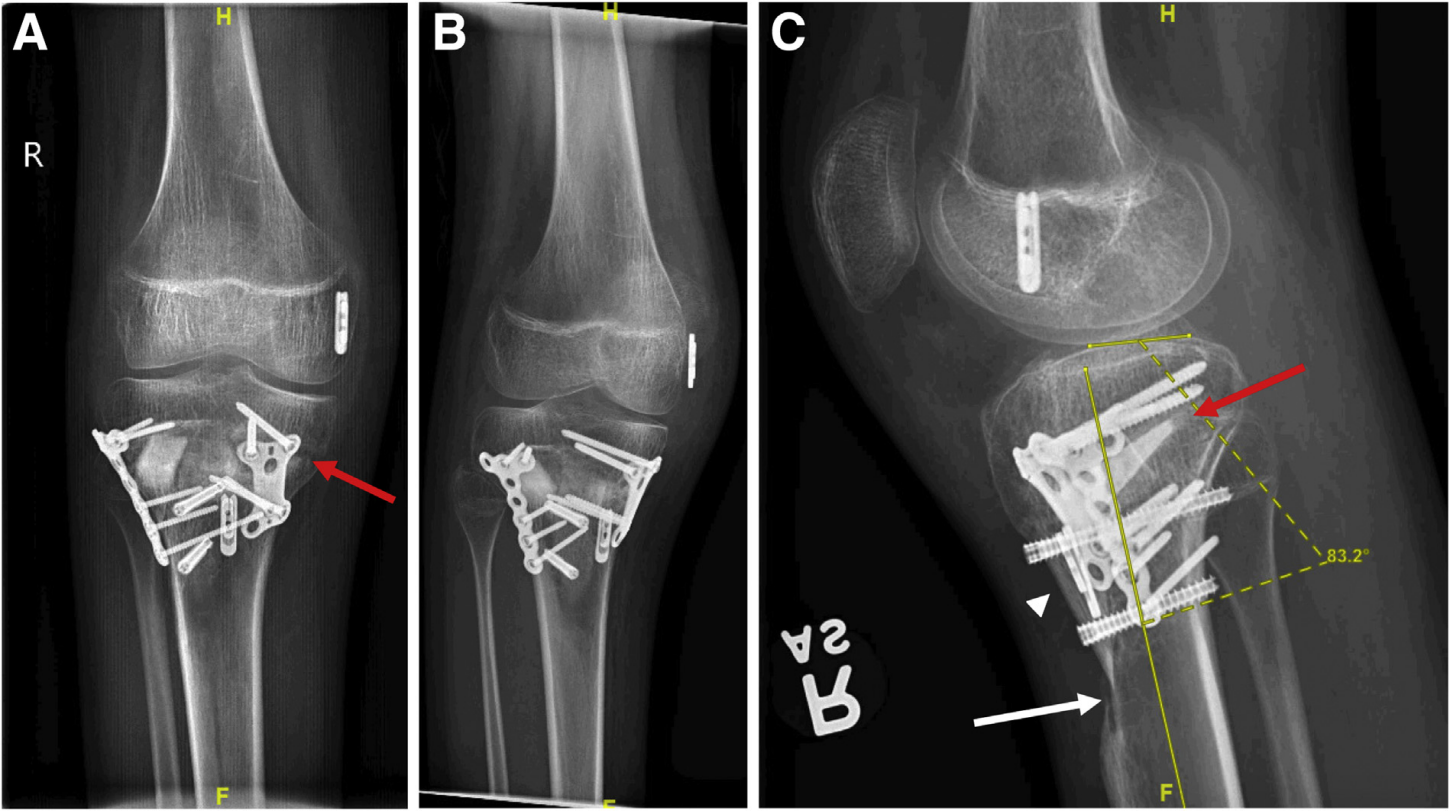
Six-week postoperative radiographs after combined high tibial osteotomy (HTO), tibial tubercle osteotomy (TTO), and posterior cruciate ligament reconstruction (PCL-R). Anteroposterior (A), oblique (B), and lateral (C) radiographs of the knee demonstrate a healed anterior opening wedge HTO and TTO with robust callus formation (red arrows) at the osteotomy site 6 weeks postoperatively. On the lateral radiograph (C), the tibial tubercle is proximal to its original location relative to the tibial diaphysis, as evidenced by the cortical defect (white arrowhead), in order to prevent patellar baja. The postoperative tibial slope is 7°, indicating that a 10° correction has been obtained.

Postoperatively, the patient is kept non-weightbearing for 6 weeks in a hinged knee brace locked in extension. Straight leg raises and ankle pump exercises are started on postoperative day 1. Full range of motion in the unlocked brace, including active and passive range-of-motion exercises, is allowed at 2 weeks postoperatively, and formal physical therapy is started at this time. At 6 weeks postoperatively, if there is radiographic evidence of union (Fig 7), weight-bearing is advanced to full weight-bearing as tolerated, and the brace is weaned at this time.

## Discussion

This article reports on the use of a combined aOW-HTO with a concomitant TTO to address chronic PCL deficiency in the setting of flat or reduced PTS. The two main previously described methods used either a supratubercle or infratubercle osteotomy. Supratubercle HTO’s have reported successful results but can alter patellar height and patellofemoral mechanics,^8^ which can lead to pain and decreased satisfaction. Infratubercle HTO’s can provide more rigid fixation but have the potential for delayed union due to cortical bone involvement.^9^ One advantage of the technique described in this article is that it allows for a significant increase in PTS, while minimizing any change in patellar height, which can lead to patellofemoral pain and other symptoms.^8^ Limitations of this technique compared to supratubercle or infratubercle HTO’s include the potential for interference between the osteotomy screws and the tibial tunnel, delayed union, and tubercle fracture.^10^

There is no established cut-off for the amount of slope necessitating a concomitant osteotomy. Bernhardson et al. reported a mean PTS of 5.7 in patients with PCL injury compared to 8.6 in patients without PCL injury.^7^ Weiler et al. reported on a series of 6 patients undergoing aOW-HTO for chronic PCL deficiency with a mean preoperative PTS of 3.7° and mean postoperative PTS of 11.5°.^5^ Of the four patients who had longer than 1 year follow up, two did not require any further surgery, and two underwent additional PCL-R. Consequently, we recommend considering a slope-increasing osteotomy in patients undergoing primary PCL-R with PTS less than 5° and, in the revision setting, with PTS less than 7°. The decision to perform a PCL-R can be made intraoperatively after osteotomy fixation, or PCL-R can be delayed and performed if patients continue to experience symptomatic PCL deficiency. Given significant rates of residual posterior laxity despite numerous advances in PCL-R techniques, the use of slope-increasing osteotomies represents an exciting new development with potential to significantly improve the treatment of PCL deficiency.

## Supplementary Data

ICMJE author disclosure forms

Video 1 This video demonstrates the technique for performing a combined tibial tubercle osteotomy (TTO), anterior opening wedge high tibial osteotomy (aOW-HTO), and posterior cruciate ligament reconstruction (PCL-R) for addressing posterior cruciate ligament (PCL) insufficiency in the setting of reduced posterior tibial slope in a right knee. This patient is a 16-year-old male who presented with right knee pain and instability 2 years after a bike injury. Further workup, including plain radiographs and magnetic resonance imaging, was notable for PCL insufficiency and reduced tibial slope. This patient was subsequently indicated for combined aOW-HTO, TTO, and PCL-R. The patient is positioned supine on a radiolucent table, and examination under anesthesia is performed. Diagnostic arthroscopy through the anterolateral portal of the knee is first performed to evaluate the status of the cruciate ligaments, menisci, and articular cartilage. A 10-cm anterior incision starting just proximal to the tibial tubercle is made, and medial and lateral skin flaps are raised. The patellar tendon is freed from the medial and lateral capsule, and a straight clamp is used to define the proximal aspect of the tibial tubercle. A line is marked 5 cm distal to the proximal aspect of the tibial tubercle, and a flat TTO is performed to yield an osteotomized tibial tubercle fragment that is ∼5 cm in length. Two guidewires are then inserted to template the planned HTO. The HTO is initially performed with an oscillating saw, and an osteotome is used to complete the osteotomy under fluoroscopic visualization to avoid inadvertent violation of the posterior tibial cortex. The osteotomy site is then opened to obtain the desired correction. The osteotomy gap is measured, and a wedge of iliac crest bone allograft is shaped and inserted into the osteotomy site, which is then further filled with further bone graft substitutes, including a preshaped block of calcium phosphate cement and demineralized bone matrix. Fixation of the tibial osteotomy is performed with medial and lateral low-profile titanium plates (Stryker, Kalamazoo, MI). The tibial tubercle fragment is then provisionally fixed to the proximal tibia, using fluoroscopy to confirm that patellar height has not been changed from preoperative patellar height. Once the tibial tubercle position is satisfactory, two headless compression screws (Arthrex, Naples, FL) are used for fixation of the tibial tubercle fragment. Arthroscopic all-inside posterior cruciate ligament reconstruction is then performed. The PCL remnant is debrided, and the posteromedial portal is established using modified Seldinger technique. The posterior capsule is elevated from the posterior tibia until the fibers of the popliteus are visualized. Using a tibial PCL guide (Arthrex), the surgeon advances the guide wire to the insertion of the PCL on the posterior tibia, ∼10 mm distal to the articular surface, and an 11-mm tibial socket is back-reamed. After a 1-cm incision is made over the medial femoral condyle, using a femoral PCL guide (Arthrex), a guidewire is advanced to the femoral origin of the PCL on the lateral aspect of the medial femoral condyle, and an 11-mm femoral socket is back-reamed. The femoral end of a previously prepared quadruple-stranded hamstring tendon allograft with preloaded suspensory buttons (Arthrex) is pulled into the femoral tunnel, and the suspensory button is flipped. The tibial end of the graft is then pulled into the tibial tunnel, and the second suspensory button is flipped. The knee is placed at 90° of flexion, an anterior drawer is applied, and the graft is subsequently tensioned on both the femoral and tibial sides.
